# *Prunus domestica* L. Fruit Extract Mitigates the Depletion of Rat Hippocampal Neurogenesis and Memory Deficits Observed in a D-Galactose-Induced Aging Rat Model

**DOI:** 10.3390/nu18193178

**Published:** 2026-09-26

**Authors:** Salinee Naewla, Puncharatsm Pannin, Papatchaya Sintow, Ram Prajit, Nataya Sritawan, Apiwat Sirichoat, Rawiwan Charoensup, Peter Wigmore, Jariya Umka Welbat

**Affiliations:** 1 Department of Basic Medical Science, Faculty of Medicine Vajira Hospital, Navamindradhiraj University, Bangkok 10300, Thailand; salinee.nae@nmu.ac.th; 2 Neurogenesis Research Group, Department of Anatomy, Faculty of Medicine, Khon Kaen University, Khon Kaen 40002, Thailand; ppuncharatsm@kkumail.com (P.P.); prapatchaya.pao@mfu.ac.th (P.S.); rampra@kku.ac.th (R.P.); natasr@kku.ac.th (N.S.); apiwsi@kku.ac.th (A.S.); 3 Department of Anatomy, Faculty of Medicine, Khon Kaen University, Khon Kaen 40002, Thailand; 4 School of Integrative Medicine, Mae Fah Luang University, Chiang Rai 57100, Thailand; rawiwan.cha@mfu.ac.th; 5 Medicinal Plant Innovation Center of Mae Fah Luang University, Mae Fah Luang University, Chiang Rai 57100, Thailand; 6 School of Life Sciences, Medical School, Queen’s Medical Centre, University of Nottingham, Nottingham NG7 2RD, UK; wigmore.peter@hotmail.com

**Keywords:** aging, memory, hippocampal neurogenesis, D-galactose, *Prunus domestica* L.

## Abstract

Background/Objectives: Accumulation of oxidative damage and brain inflammation plays a role in aging-associated memory impairment. D-galactose (D-gal) is a monosaccharide used to induce aging in rat models by promoting oxidative damage and neuroinflammation. *Prunus domestica* L. (PD) contains high levels of flavonoid compounds, which possess antioxidant, anti-inflammatory, neuroprotective and anti-aging properties. This study investigated the effects of PD fruit extract on memory, vascular-associated cell proliferation, immature neuronal cells, and survival of neurons in the dentate gyrus (DG) using a rat model of D-gal-induced aging. Methods: Forty-eight male Sprague–Dawley (SD) rats, two months old, were randomly assigned to eight groups: vehicle (0.9% normal saline), D-gal (50 mg/kg), PD (75, 100, and 150 mg/kg), or D-gal (50 mg/kg) co-administered with PD (75, 100 or 150 mg/kg). Spatial memory was assessed using the novel object location (NOL) test. Hippocampal neurogenesis and vascular-associated cell proliferation in the DG were evaluated using immunofluorescence staining for the co-localization of the markers Ki-67/rat endothelial cell antigen-1 (RECA-1) and 5-bromo-2′-deoxyuridine/neuronal nuclear protein (BrdU/NeuN), as well as doublecortin (DCX). Results: Rats treated with D-gal alone did not show a significant preference for the novel location. Additionally, D-gal treatment reduced the numbers of Ki-67-, Ki-67/RECA-1-, BrdU/NeuN-, and DCX-stained cells in the hippocampus. Conversely, co-administration of PD fruit extract was associated with a significant preference for the novel location, and this treatment also attenuated the D-gal-triggered reductions in cellular markers. Conclusions: These finding suggest that PD fruit extract exerts a neuroprotective effect against D-gal-induced brain aging, potentially preserving spatial memory-related performance in rats.

## 1. Introduction

Aging is a natural process that leads to progressive physiological decline of the organism and an increasing risk of age-related conditions, including cancer and metabolic, cardiovascular, and neurodegenerative diseases [1]. The rapidly aging global population and its associated brain aging present a worldwide challenge, primarily due to rising rates of disability and disruption of daily life. Brain aging is characterized by neuronal loss, reduced brain volume, and synaptic deterioration, all of which contribute to cognitive decline and dementia [2]. Numerous studies have reported that brain aging is associated with the overproduction of reactive oxygen species (ROS) and a decline in antioxidant enzymatic activity [3,4], leading to oxidative stress, neuroinflammation, and apoptosis [5,6,7]. In addition, ROS can induce memory impairment mediated by alterations in hippocampal synaptic plasticity and neural development [8,9]. Previous studies have shown that neural regeneration in the subgranular zone (SGZ) of the dentate gyrus (DG) within the hippocampus is related to learning and memory functions [10,11,12]. This area generates young neurons originating from neural stem cells (NSCs) or neural progenitor cells (NPCs) throughout adulthood [13,14]. Mature newborn neurons exhibit gene expression patterns associated with hippocampal circuit function and memory processes [15]. Adult-born neurons in the SGZ integrate into functional hippocampal circuits and contribute to hippocampal plasticity and memory-related processes. Consequently, impairment of adult hippocampal neurogenesis directly contributes to deficits in hippocampus-dependent learning and memory [16,17]. To evaluate these dynamic cellular processes, stage-specific markers are required. Ki-67, an endogenous nuclear marker expressed during active cell-cycle phases, is therefore a well-established marker of cell proliferation within the adult SGZ [18]. Ki-67/rat endothelial cell antigen-1 (RECA-1) is an endothelial cell marker that can be used in combination with Ki-67 to identify proliferating vascular endothelial cells within the hippocampal DG [19]. In addition, doublecortin (DCX) and 5-bromo-2′-deoxyuridine/neuronal nuclear protein (BrdU/NeuN) labeling were used as complementary markers representing developing immature neurons and newly generated neuronal cells, respectively [20,21].

D-galactose (D-gal) is a simple monosaccharide commonly found in various foods, including fruits and vegetables [22,23]. However, the overaccumulation of D-gal induces systemic toxicity. Specifically, D-gal has been reported to trigger the overproduction of ROS [7]. Chronic administration of D-gal in animal models has been shown to induce an accelerated brain aging phenotype characterized by several features associated with brain aging, including cognitive impairment and hippocampal dysfunction [6,24]. The D-gal-induced aging rat model is widely used to investigate age-related cognitive decline, hippocampal dysfunction, and impaired hippocampal neurogenesis, as it represents several key features of brain aging [6,25]. D-gal is reduced by galactose reductase to form galactitol, which induces osmotic stress and disrupts mitochondrial function. These disturbances induce oxidative stress and inflammatory activation, ultimately leading to apoptosis and degenerative changes that promote aging and age-related disorders [6,26]. Interestingly, previous studies have demonstrated that D-gal administration impairs neurogenesis, neuronal migration, and the survival of newly generated neurons in the SGZ of the DG [20,27]. Similarly, earlier research has shown that a D-gal dose of 50 mg/kg/day induces memory impairment in rats [20,28]. Together, these findings indicate that D-gal induces hippocampal neurodegeneration and memory impairment, exhibiting several features associated with accelerated brain aging in animals [27].

*Prunus domestica* L. (PD), commonly known as the European plum, belongs to the genus Prunus within the Rosaceae family [29,30,31]. Its trees are medium-sized, averaging 8–10 m in height, and are found in temperate climate regions worldwide. PD contains high levels of phenolic and flavonoid compounds that contribute to the maintenance of health and physiological functions [29,30]. Studies have shown that PD exhibits pharmacological effects, including anticancer, anti-inflammatory, potent antioxidant and anti-aging properties [29,32]. Moreover, the antioxidant capacity detected in PD is involved in neurological action [33,34]. Furthermore, the same study reported that PD administration enhances learning and memory [33]. In addition, recent evidence has demonstrated the neuroprotective potential of PD in aging models. Oral supplementation with PD fruit extract significantly improved recognition memory, increased hippocampal cell proliferation and enhanced antioxidant capacity in D-gal-induced aging rats [35]. However, the previous study primarily focused on recognition memory and cell proliferation, while other stages of hippocampal neurogenesis and the effects of PD on hippocampus-dependent spatial memory remain unclear in the D-gal-induced rat model.

Therefore, the present study aimed to investigate the effects of PD fruit extract on spatial memory and multiple stages of hippocampal neurogenesis including vascular-associated proliferation, neuronal differentiation and neuronal survival in a D-gal-induced aging model. Learning and memory abilities were assessed using the novel object location (NOL) test. Adult neurogenesis in the SGZ of the hippocampal DG was assessed using Ki-67/RECA-1, BrdU/NeuN, and DCX staining.

## 2. Materials and Methods

### 2.1. Animals

Forty-eight male Sprague–Dawley (SD) rats (8 weeks old and 280–300 g body weight) were supplied by Nomura Siam International Co., Ltd., Pathumwan, Bangkok, Thailand. The sample size (n = 6 per group; 8 groups in total) was selected based on an effect size determined from our previous study using the same D-gal-induced aging model and PD fruit extract [35]. The experimental unit was an individual rat, which was randomly assigned to a treatment group and used as the unit of analysis. No predefined exclusion criteria were established, and no animals or data points were excluded from the analyses. All groups consisted of 6 rats and were included in the final analyses.

The rats were accommodated four per plastic cage at the Northeast Laboratory Animal Center, Khon Kaen University, and acclimatized for four weeks before the investigation. All rats were held under 12 h light and dark periods at 23 ± 2 °C with continuous availability of food and water. No specific environmental enrichment was provided. Rats were monitored daily for general health status and signs of distress throughout the experimental period. No adverse events occurred, and specific humane endpoints were established by monitoring the animals’ weight daily. All investigative methodologies were authorized by the Institutional Animal Care and Use Committee of Khon Kaen University (Approval Nos. IACUC-KKU-49/64 and IACUC-KKU (C)-47/69).

### 2.2. Drug Administrations

Rats were randomly assigned to eight groups. Group I (vehicle; VH) was given 0.9% normal saline via intraperitoneal administration and distilled water orally for 8 weeks. Group II (D-gal) received D-gal (50 mg/kg, Sigma-Aldrich, St. Louis, MO, USA) via intraperitoneal administration for 8 weeks. Group III (PD 75), Group IV (PD 100), and Group V (PD 150) received PD fruit extract orally for 8 weeks at doses of 75, 100, and 150 mg/kg, respectively. Group VI (D-gal + PD 75), Group VII (D-gal + PD 100), and Group VIII (D-gal + PD 150) received D-gal (50 mg/kg) via intraperitoneal administration and PD fruit extract orally for 8 weeks at doses of 75, 100, and 150 mg/kg. The D-gal administration protocol was selected to induce brain aging-associated alterations, while the doses of the PD fruit extract were selected based on previous studies demonstrating potential neuroprotective effects in experimental models [35].

At the onset of the treatment period, all rats were received an intraperitoneal injection of BrdU (250 mg/kg, Sigma Aldrich, USA) suspended in 0.9% normal saline. BrdU administration was performed to label newly generated cells, enabling the assessment of newborn cell survival in the hippocampus [20].

### 2.3. Plant Collection and Extraction

Fresh PD fruits were collected from Mae Chan District, Chiang Rai Province, Thailand (20°11′57″ N/99°53′5″ E), in late February 2021. The PD fruit samples used in this study were not assigned to a specific cultivar. The voucher specimen was authenticated and preserved at the Medicinal Plants Innovation Center, Mae Fah Luang University, Thailand. Briefly, the PD fruit extract was prepared by maceration using 95% (*v*/*v*) ethanol for a duration of 7 days, following the protocol previously described by Aranarochana et al. (2025) [35]. The extract was subsequently separated through a filter paper and vaporized using a rotary evaporator. The extraction yield of the PD fruit extract was 4.84% (*w*/*w*). This extract represents the same batch fully characterized in our previous study, which established its phytochemical profile, including total phenolics, total flavonoids, tannins, and anthocyanins. UHPLC–QTOF–MS analysis identified 17 compounds in the extract, including phenolic compounds, xanthone-related compounds, terpenoid-related compounds, fatty acids, and other metabolites. Based on this phytochemical profile and the reported biological properties of these phytochemical groups, phenolic and flavonoid constituents may contribute to the biological effects of the PD fruit extract [35]. Nevertheless, the specific contributions of individual compounds to these effects have yet to be elucidated.

For animal administration, the dried PD fruit extract was freshly dissolved in distilled water daily to prepare dosing solutions at concentrations of 75, 100, and 150 mg/mL, corresponding to doses of 75, 100, and 150 mg/kg BW, respectively, when administered to rats via oral gavage at a volume of 1 mL/kg BW. The solutions were administered once daily for 8 consecutive weeks.

### 2.4. Behavioral Assessment

#### NOL Test

The NOL test was conducted to evaluate hippocampus-dependent spatial learning. This provided a behavioral measurement of cognitive impairment associated with D-gal-induced brain aging and allowed the assessment of the effects of PD fruit extract on memory function in rats [36,37]. The NOL test was conducted following treatment. Rats were introduced to an empty field arena for acclimatization (50 × 50 × 50 cm) for 30 min one day before the test. The NOL test comprised two examinations: familiarization and choice trials. During the familiarization trial, rats were arranged in the same arena and given the freedom to inspect a matched set of objects placed at different positions (location A and location B) for 3 min. Following exploration, rats were moved back to animal housing for a quarter hour. The arena and objects were diligently sanitized with a 20% ethanol mixture to eradicate any smell signals. In the choice trial, rats were permitted to investigate one object at the identical position (familiar location; FL) and the alternative object at a new position (novel location; NL) for 3 min. During each trial, the distance moved and exploration time were documented using the EthoVision program (EthoVision^®^, XT version 12, Noldus, Wageningen, Netherlands). The exploration time was noted when the rat’s nose was no more than 2 cm from the object. Based on the exploration time in the choice trial, the preference index (PI) was evaluated as previously reported [36,38,39]. The NOL test was conducted without investigator blinding to treatment allocation. All behavioral sessions were video-recorded and retrospectively analyzed using predefined, standardized scoring criteria.

### 2.5. Hippocampal Neurogenesis Assessment

#### Tissue Preparation

After completing behavioral testing, all rats were sacrificed by immediate stunning, followed by neck separation. The whole brains were then promptly excised and divided into two hemispheres. Each hemisphere was transferred to a 30% sucrose solution, enclosed in an optimal-cutting-temperature compound (Thermo Fisher Scientific, Darmstadt, Germany) and frozen in isopentane cooled by liquid nitrogen (Sigma Aldrich, St. Louis, MO, USA). The brains were stored at −80 °C and then coronally sectioned between Bregma −2.8 and −6.3 mm with a thickness of either 20 or 40 μm using a cryostat (HM550 Cryostat Series, A.S. Science Co. Ltd., Walldorf, Germany).

### 2.6. Immunofluorescence Labeling

Vascular-associated cell proliferation was assessed using Ki-67/RECA-1 double immunofluorescence staining [40]. The brain sections (20 μm thickness) were preserved with 0.5% paraformaldehyde before being incubated with the primary antibody Ki-67 (NOVOCASTRA, Newcastle, UK; 1:150) and the secondary antibody Alexa Fluor 488 IgG (Invitrogen, Waltham, MA, USA, A11059; 1:250). After that, sections were exposed to the primary monoclonal mouse RECA-1 antibody (Santa Cruz Biotechnology, Dallas, TX, USA; 1:100) before being exposed to a secondary antibody, Alexa Fluor 568 IgG (Invitrogen, Waltham, MA, USA, A11077; 1:250), and contrast staining with diamidinophenylindole (DAPI) was performed (Molecular probes, Eugene, OR, USA; 1:6000).

Immature neurons were assessed using DCX immunofluorescence staining [21]. Sections (40 μm thickness) were exposed to the primary antibody anti-DCX (1:100, Santa Cruz, USA) before being exposed to a secondary antibody, Alexa Fluor 488 IgG (1:500, Molecular probes, Eugene, OR, USA). At last, the sections were counterstained with propidium iodide (1:6000, Sigma Aldrich, St. Louis, MO, USA).

The survival of newborn neurons was assessed using BrdU/NeuN double immunofluorescence staining [20]. Sections (40 μm thickness) were exposed to the primary anti-BrdU antibody (1:200, Abcam, Cambridge, UK), followed by the secondary antibody Alexa Flour 568 IgG (1:200, Invitrogen, Carlsbad, CA, USA). Then, the sections were exposed to the primary antibody anti-NeuN (1:500, Abcam, Cambridge, UK), followed by the secondary antibody Alexa Fluor 488 IgG (1: 500, Invitrogen, Carlsbad, CA, USA). At last, the sections were contrast-stained with propidium iodide (1:6000, Sigma Aldrich, St. Louis, MO, USA).

### 2.7. Microscopic Quantification

The overall counts of Ki-67-, Ki-67/RECA-1-, BrdU/NeuN-, and DCX-stained cells were determined utilizing a fluorescence microscope (Nikon Eclipse Ci-L, Nikon Instruments, Inc., Melville, NY, USA). Nine sections, which were chosen from every 15th section covering the full DG, were stained to assess Ki-67 and Ki-67/RECA-1-stained cells, whereas every 8th section was stained to probe DCX- and BrdU/NeuN-stained cells. Ki-67- and DCX-stained cells were quantified, encompassing three cell distances in the SGZ, while BrdU/NeuN-stained cells were quantified within the SGZ and a granular cell layer of the DG. Ki-67-stained cells within twice the diameter of a cell of a RECA-1-stained cell were sorted as vascular-associated Ki-67-stained cells. The results of Ki-67 and Ki67/RECA-1 staining were amplified by a factor of 15, while those for DCX- and BrdU/NeuN-stained cells were amplified by a factor of 8 [38,39,41]. Microscopic quantification of immunofluorescence-stained cells was performed without investigator blinding to treatment allocation. Nevertheless, predefined image acquisition settings and standardized cell-counting criteria were consistently applied across all experimental groups to minimize potential observer bias.

### 2.8. Statistical Analysis

All findings were presented as the mean ± standard error of the mean (SEM). For the NOL test, locomotor activity data were analyzed using one-way ANOVA followed by Bonferroni’s post hoc test. Exploration times between the familiar and novel locations were compared using paired Student’s *t*-tests. PI values were evaluated against the 50% chance level using a one-sample *t*-test.

All datasets were assessed for normality and homogeneity of variance before parametric statistical analyses. Data from the immunofluorescence analyses were analyzed by two-way ANOVA to evaluate the main effects of D-gal administration, PD fruit extract treatment and their interaction, followed by Bonferroni’s multiple comparison test using GraphPad Prism (Version 9.0; GraphPad Software Inc., San Diego, CA, USA). Statistical significance was set at *p* < 0.05.

## 3. Results

### 3.1. Impacts of D-Gal and PD Fruit Extract on Memory Impairment

The procedure for the NOL test is illustrated in Figure 1A. Locomotor activity in the NOL test was evaluated through the measurement of the distance traveled during the habituation trial. No statistically significant differences in distance traveled were observed among the groups (F(7, 40) = 2.139, *p* = 0.0613, one-way ANOVA, Table 1). All rats showed no statistically significant difference in exploration time between the objects in locations A and B during the familiarization trial (*p* > 0.05, paired Student’s *t*-test, Figure 1B). In the choice trial, rats in the vehicle, PD and co-administration (D-gal and PD fruit extract) groups spent more time exploring the object in the NL than the object in the FL (*p* < 0.05 and *p* < 0.01, paired Student’s *t*-test, Figure 1C). However, this difference was not observed in the D-gal group (*p* > 0.05, paired Student’s *t*-test, Figure 1C). PI values were significantly greater than the 50% chance level in the vehicle, PD, and co-administration (D-gal + PD fruit extract) groups (VH: *p* < 0.01; PD 75, 100, and 150 mg/kg: *p* < 0.05 for all; D-gal + PD 75, 100, and 150 mg/kg: *p* < 0.001, *p* < 0.01, and *p* < 0.05, respectively; one-sample *t*-test, Table 2 and Figure 2), demonstrating a significant preference for the novel location. By contrast, this preference was not observed in the D-gal group (*p* > 0.05, one-sample *t*-test, Table 2 and Figure 2), in which PI values did not differ significantly from the chance level.

### 3.2. Impacts of D-Gal and PD Fruit Extract on Vascular-Associated Cell Proliferation

The data regarding the impact of D-gal and PD fruit extract on the total number of Ki-67-stained cells, vascular-associated Ki-67-stained cells and non-vascular-associated Ki-67-stained cells in all groups are displayed in Table 3 and Figure 3 and Figure 4. Two-way ANOVA revealed significant main effects of D-gal administration (F(1,40) = 52.39, η^2^p = 0.567, *p* < 0.0001) and PD fruit extract dose (F(3,40) = 117.6, η^2^p = 0.898, *p* < 0.0001). In addition, a significant interaction between D-gal and PD fruit extract dose was observed (F(3,40) = 14.35, η^2^p = 0.518, *p* < 0.0001), indicating that the effect of PD fruit extract treatment depended on D-gal exposure. A Bonferroni multiple comparison test revealed a marked decrease in the sum of Ki-67-stained cells in the D-gal group compared to the vehicle group (*p* < 0.0001). The sum of Ki-67-stained cells of the co-treatment (D-gal and PD fruit extract) groups also markedly exceeded that in the D-gal group (PD fruit extract doses at 75, 100, and 150 mg/kg; all *p* < 0.0001). These results indicate that D-gal markedly suppressed total proliferative activity, whereas PD treatment significantly attenuated this suppression.

The count of vascular-associated Ki-67-stained cells was analyzed using a two-way ANOVA, which revealed significant main effects of D-gal administration (F(1,40) = 71.36, η^2^p = 0.641, *p* < 0.0001) and PD fruit extract dose (F(3,40) = 231.3, η^2^p = 0.945, *p* < 0.0001), as well as a significant interaction between D-gal and PD fruit extract dose (F(3,40) = 25.43, η^2^p = 0.656, *p* < 0.0001). This significant interaction indicates that the effect of PD fruit extract treatment differed according to D-gal exposure. Bonferroni’s multiple comparison test demonstrated that rats exposed to D-gal exhibited a marked decrease in vascular-associated Ki-67-stained cells compared to the vehicle group (*p* < 0.0001). Furthermore, within the D-gal condition, PD fruit extract treatment at 75, 100, and 150 mg/kg resulted in significantly higher cell counts than the D-gal group (*p* < 0.0001, *p* < 0.0001 and *p* < 0.0001, respectively). These findings indicate that D-gal markedly suppressed vascular-associated proliferative activity, whereas PD fruit extract treatment significantly attenuated this reduction.

For the count of non-vascular-associated Ki-67-stained cells, the two-way ANOVA showed significant main effects of D-gal administration (F(1,40) = 18.79, η^2^p = 0.320, *p* < 0.0001) and PD fruit extract dose (F(3,40) = 26.71, η^2^p = 0.667, *p* < 0.0001), as well as a significant interaction between D-gal and PD fruit extract dose (F(3,40) = 3.56, η^2^p = 0.211, *p =* 0.0224), indicating that the effect of the PD fruit extract depended on D-gal exposure. The Bonferroni multiple comparison test demonstrated that the count of non-vascular-associated Ki-67-stained cells in the vehicle group was significantly higher than that in the D-gal group (*p =* 0.0004). However, the count of non-vascular-associated Ki-67-stained cells in the D-gal + PD groups treated with PD at 75, 100, and 150 mg/kg was considerably higher than that in the D-gal group (all *p* < 0.0001). These results imply that co-administration with PD fruit extract enhanced non-vascular-associated cell proliferation in the hippocampus of rats exhibiting D-gal-induced aging.

### 3.3. Impacts of D-Gal and PD Fruit Extract on Developing Neuronal Cells

The data on the impact of D-gal and PD fruit extract on developing neuronal cells are presented in Table 4 and Figure 5. Two-way ANOVA revealed significant main effects of D-gal administration (F(1, 40) = 39.69, η^2^p = 0.498, *p* < 0.0001) and PD fruit extract dose (F(3, 40) = 29.09, η^2^p = 0.686, *p* < 0.0001). Additionally, a significant interaction between PD fruit extract dose and D-gal exposure was detected (F(3, 40) = 5.82, η^2^p = 0.304, *p =* 0.0021), suggesting that the effect of PD fruit extract varied depending on D-gal treatment.

The Bonferroni multiple comparison test showed that the count of DCX-stained cells in the D-gal group was also markedly lower compared to that in the vehicle group (*p* < 0.0001). In D-gal-treated rats, PD fruit extract at 75, 100, and 150 mg/kg significantly increased the number of DCX-stained cells compared with that in the D-gal group (all *p* < 0.0001). These observations imply that D-gal decreases the count of developing neurons, while co-administration with doses of PD fruit extract can ameliorate the decline in immature neurons produced by D-gal in the SGZ.

### 3.4. Impact of D-Gal and PD Fruit Extract on Neuronal Cell Survival

The data on the impact of D-gal and PD fruit extract on neuronal cell survival are presented in Table 4 and Figure 6. Two-way ANOVA showed significant main effects of D-gal administration (F(1, 40) = 61.84, η^2^p = 0.607, *p* < 0.0001) and PD fruit extract dose (F(3, 40) = 32.98, η^2^p = 0.712, *p* < 0.0001). Additionally, a significant interaction between D-gal and PD fruit extract dose (F(3, 40) = 14.48, η^2^p = 0.521, *p* < 0.0001) was detected, indicating that the effect of PD fruit extract varied depending on D-gal exposure.

The Bonferroni multiple comparison test revealed a significant reduction in the count of BrdU/NeuN-stained cells in the D-gal group compared to that in the vehicle group (*p* < 0.0001). Conversely, recovery was observed in the groups co-treated with PD fruit extract (75, 100, 150 mg/kg: *p* < 0.0001, *p* < 0.0001, and *p* = 0.0007, respectively) when compared to the D-gal group. The outcomes indicated that D-gal suppressed neuronal cell survival, whereas treatment with PD fruit extract significantly attenuated this suppression and is associated with increased survival of newborn neurons in D-gal-treated rats.

## 4. Discussion

Aging causes alterations in brain, vasculature, and memory functions. It is widely recognized that brain aging is often seen with decreased neuronal generation, which triggers impairments in learning and memory. Memory impairments are the most disruptive to daily activities and are associated with neurodegenerative disorders, including Alzheimer’s disease (AD) [42]. In the current investigation, D-gal-induced reductions in neurogenesis were accompanied by an absence of significant novel-location preferences, findings that were consistent with impaired spatial memory-related performance in this aging model. However, co-administration of PD fruit extract with D-gal at doses of 75, 100, 150 mg/kg was associated with a significant preference for the novel location and attenuated the D-gal-induced reductions in neurogenesis.

In the current investigation, the NOL test, which is widely used to assess hippocampus-dependent spatial learning and memory [36,37], was employed to evaluate the effects of PD fruit extract and D-gal on memory in rats. Locomotor activity, as evaluated by distance moved during the habituation trial, showed no statistically significant differences among treatment groups, indicating that group differences in the NOL test were not attributable to differences in general motor activity. In general, rats spend significantly more time inspecting an object placed in a novel location when measured against an object in a familiar location [43]. However, rats exposed to D-gal alone did not show this preference for the novel location, indicating impaired spatial memory. These data are consistent with prior studies, which confirmed D-gal-triggered learning and memory impairments by demonstrating that D-gal-treated rats failed to recognize an object in a novel location in the NOL test. Previous studies have demonstrated that rats treated with D-gal (50 mg/kg) for 8 weeks revealed impaired cognition in the NOL and novel object recognition (NOR) tests [20,28]. By contrast, rats that received PD fruit extract alone (75, 100, and 150 mg/kg) or co-treatment with D-gal and PD fruit extract (75, 100, and 150 mg/kg) showed a significant preference for the novel location. These findings are consistent with earlier reports regarding the cognitive-enhancing effects of PD extract at doses of 75, 100, and 150 mg/kg [33]. Additionally, a previous study demonstrated that PD improves recognition memory and exhibits strong antioxidant properties, including the restoration of superoxide dismutase (SOD) activity in D-gal-treated rats. PD is also found to contain abundant phenolics, tannins, flavonoids, and anthocyanins, which contribute significantly to its antioxidant properties [35]. These effects likely stem from D-gal-induced accelerated aging, which impairs memory through mechanisms such as oxidative stress, inflammation and hippocampal damage. The restoration of memory—as evidenced by a significant novel-location preference in the PD and D-gal + PD groups—counters this decline. These results align with previous reports demonstrating that plum juice enhances working memory in aging rats, as shown by improved performance in the Morris water maze (MWM) [32].

It has been mentioned that memory malfunction is influenced by a decline in neuron count in the hippocampus [44,45]. Both in animals and humans, a diminution of the number or size of neurons is correlated with an aging hippocampus [46]. Changes in neurogenesis and brain aging are linked to learning and memory processes [47,48]. Neurogenesis is the endogenous process that encompasses the proliferation and development of newborn neuronal cells [45]. Therefore, we assessed the impact of D-gal and PD fruit extract on neurogenesis in the SGZ of the hippocampal DG using Ki-67/RECA-1 to assess cell proliferation and vascular endothelial cells. BrdU/NeuN was used to evaluate cell survival and neuronal association, and DCX was used to identify immature neurons.

For vascular-associated cell proliferation, Ki-67, a protein localized in the nucleus, is present in cells undergoing division throughout all phases of the ongoing cell cycle, encompassing the G1, S, G2, and M phases [40]. Therefore, it is widely used as a marker for cell proliferation [49]. RECA-1 is an endothelial cell antigen associated with blood vessels [19]. The vasculature is fundamental to the division and differentiation of NSCs. Endothelial cells in blood vessels can regulate brain-derived neurotrophic factor (BDNF) concentrations, which consequently enhances neurogenesis and neuronal cell survival [50,51,52]. The decline in BDNF protein levels is specifically connected with learning and memory impairments and can influence behavioral changes [53]. In the ongoing investigation, rats exposed to D-gal alone displayed a substantial reduction in the total count of proliferating cells and vascular-associated proliferating cells in the hippocampus. This finding aligns with an earlier report demonstrating that D-gal induces apoptosis and diminishes the production of hippocampal RECA-1, indicating that D-gal induces neurovascular inflammation and blood–brain barrier (BBB) abnormalities [54]. By contrast, the PD (75, 100, and 150 mg/kg) and D-gal + PD (75 and 100 mg/kg) groups showed increases in the total number of proliferating cells and vascular-associated proliferating cells. This suggests that PD fruit extract may counteract the reduction in cell proliferation associated with vascular endothelial cells and triggered by D-gal administration. Consistent with previous findings, D-gal administration significantly reduced the number of Ki-67-stained cells in the DG; however, PD fruit extract co-administration markedly restored this reduction [35]. Notably, the present study further demonstrates that PD fruit extract enhances vascular-associated proliferation, as indicated by increased Ki-67/RECA-1-stained cells. This finding is particularly important, as the neurogenic niche in the hippocampus is closely associated with the vasculature, providing metabolic support and regulatory signals essential for neural stem cell function. The current results suggest that PD fruit extract not only promotes cell proliferation but also appears to maintain the integrity of the neurovascular microenvironment that supports neurogenesis. PD has been disclosed to enhance anti-inflammatory effects, with its polyphenolic compounds reducing the formation of pro-inflammatory indicators, for instance nitric oxide (NO) and cyclooxygenase-2 (COX-2). These findings suggest the potential use of PD in the management of neuronal toxicity and inflammation in vascular endothelial cells [55].

Identification of immature neurons and neuronal cell survival was achieved using DCX and BrdU/NeuN staining. DCX, a microtubule-stabilizing protein, is crucial for neuronal migration during brain development and is used as an indicator for immature neuronal cells in the brain [21,56]. BrdU is a synthetic thymidine derivative that combines with recently assembled DNA throughout the cell cycle’s S phase [57,58]. BrdU is commonly applied to detect cell survival in the brain [59]. NeuN is a neuron-specific nuclear protein found in mature neurons, making it a consistent indicator for detecting mature neurons [60]. In this study, D-gal significantly decreased the number of immature neurons and the survival of neuronal cells in the SGZ of the DG within the hippocampus. This finding aligns with earlier research revealing that D-gal negatively affects neurogenesis, as evidenced by Ki-67, DCX, and BrdU/NeuN markers [28,61,62]. A previous study reported that D-gal induces the accumulation of oxidative stress and free radicals, resulting in inflammatory responses and cell apoptosis. These processes have been linked to memory impairment [6,26,63]. D-gal induces oxidative stress, lipid peroxidation, inflammation, and elevated levels of malondialdehyde (MDA) and ROS. These effects consequently disrupt neurogenesis [61,64,65,66]. Consistently, rats receiving D-gal also exhibited a reduction in SOD, glutathione peroxidase (GSH-Px), glutathione, catalase (CAT) and total antioxidant capacity (TAOC) in the hippocampus [63,67]. Oxidative stress is aligned with the initial and progressive deterioration of cellular functions, contributing to the aging process and age-linked neurodegenerative conditions [3,4,5]. Previous reports have also postulated that D-gal induces neuronal apoptosis and reduces the survival and migration of immature neuronal cells in the GCL [27,64]. Additionally, D-gal decreases the level of calmodulin-dependent protein kinase (pCAMK) II, BDNF, and Phosphorylated cAMP Response Element-Binding (pCREB) in the hippocampus. These findings suggest that D-gal reduces adult neurogenesis, synapse formation, and active synaptic plasticity. Furthermore, D-gal can induce the production of inflammatory agents, for instance interleukin 1 beta (IL1β), tumor necrosis factor alpha (TNFα), and apoptosis-related proteins, which contribute to neuroinflammation, neurodegeneration, and neuronal apoptosis, ultimately accelerating aging [61,64,68]. Our results are consistent with the use of D-gal as an accelerated aging model characterized by several features associated with brain aging [7]. Conversely, rats treated with PD fruit extract (75, 100, and 150 mg/kg) or co-administered D-gal and PD fruit extract (75, 100, and 150 mg/kg) exhibited a reversal of the reduced levels of immature neurons and neuronal cell survival caused by D-gal. The improvement in neuronal differentiation and survival in D-gal-treated rats may be attributed to the antioxidant properties of PD. This is supported by previous findings demonstrating that PD restores SOD activity and significantly enhances antioxidant defense, thereby attenuating ROS-mediated neuronal damage and promoting hippocampal neurogenesis [35]. PD is rich in phenolic compounds, such as flavonoids, which process various pharmacological activities, including cytotoxic, anticancer, anti-inflammatory and antioxidant effects [34]. PD suppressed inflammation by inhibiting NF-kB activation, which blocks ROS generation and reduces the impacts of oxidative stress induced by hydrogen peroxide (H_2_O_2_) [34,55]. Moreover, the use of PD ethanolic extract markedly enhances learning and memory in amnesic mice by suppressing acetylcholinesterase enzyme (AChE) and reducing oxidative stress [69]. Therefore, the neuroprotective effects of PD in the D-gal-induced aging model may be partly attributed to its ability to alleviate oxidative stress and neuroinflammation. PD also contains phytochemicals known as anthocyanins, a subclass of flavonoids. Anthocyanins have been reported to enhance memory functions and scavenge superoxide radicals [32,70,71]. Interestingly, chlorogenic acids (CGAs) are phenolic compounds found in PD that may contribute to the downregulation of the TNF-α expression in lymphocytes [72]. Moreover, CGAs prevent neurodegenerative disorders and improve vascular function, as well as learning and memory functions [73,74,75]. CGAs also exert antioxidant and neuroprotective effects and promote neuronal differentiation [76,77,78].

The present study suggests that PD fruit extract improves spatial memory performance, as indicated by a significant preference for the novel location, and affects multiple stages of hippocampal neurogenesis and the microenvironment. Therefore, treatment with PD fruit extract at all doses may attenuate the D-gal-induced reductions in vascular-associated cell proliferation, immature neuron numbers and neuronal cell survival while concurrently improving performance in the novel-location preference test.

These findings suggest that the PD fruit extract is associated with preserved spatial memory-related performance and enhanced hippocampal neurogenesis in an accelerated aging model. This preservation, as indicated by a significant preference for the novel location, may occur through the antioxidant and anti-inflammatory properties of the extract. While these findings support the neuroprotective potential of PD fruit extract in age-related cognitive decline, the underlying molecular mechanisms were not directly examined in the present study and warrant further investigation. Additionally, while the PD fruit extract demonstrated beneficial effects in the D-gal-induced accelerated aging model, its translational potential for natural aging and clinical human applications remains to be determined.

Furthermore, the use of a crude 95% ethanol extract precluded determination of the specific contributions of individual bioactive constituents. Further studies using isolated compounds or standardized fractions are warranted. Finally, a limitation of this study is that behavioral testing and microscopic cell quantification were not performed under blinded conditions. Nevertheless, predefined and standardized scoring, image acquisition, and cell-counting procedures were consistently applied across all experimental groups to ensure methodological consistency and minimize potential bias.

## 5. Conclusions

In conclusion, our results suggest that the PD fruit extract may exert protective effects on hippocampal neurogenesis and may improve spatial memory-related performance in D-gal-treated aging rats. The numbers of proliferating cells co-labeled with markers of vascular endothelial cells, neuronal cell survival and immature neuronal cells were substantially diminished following D-gal administration, consistent with the impaired spatial memory-related performance described above. However, combined administration of D-gal and PD fruit extract was associated with a significant preference for the novel location and attenuated the D-gal-induced reductions in neurogenesis-related markers, consistent with preserved spatial memory-related performance. These findings support the potential neuroprotective effects of the PD fruit extract against age-related cognitive decline.

## Figures and Tables

**Figure 1:**
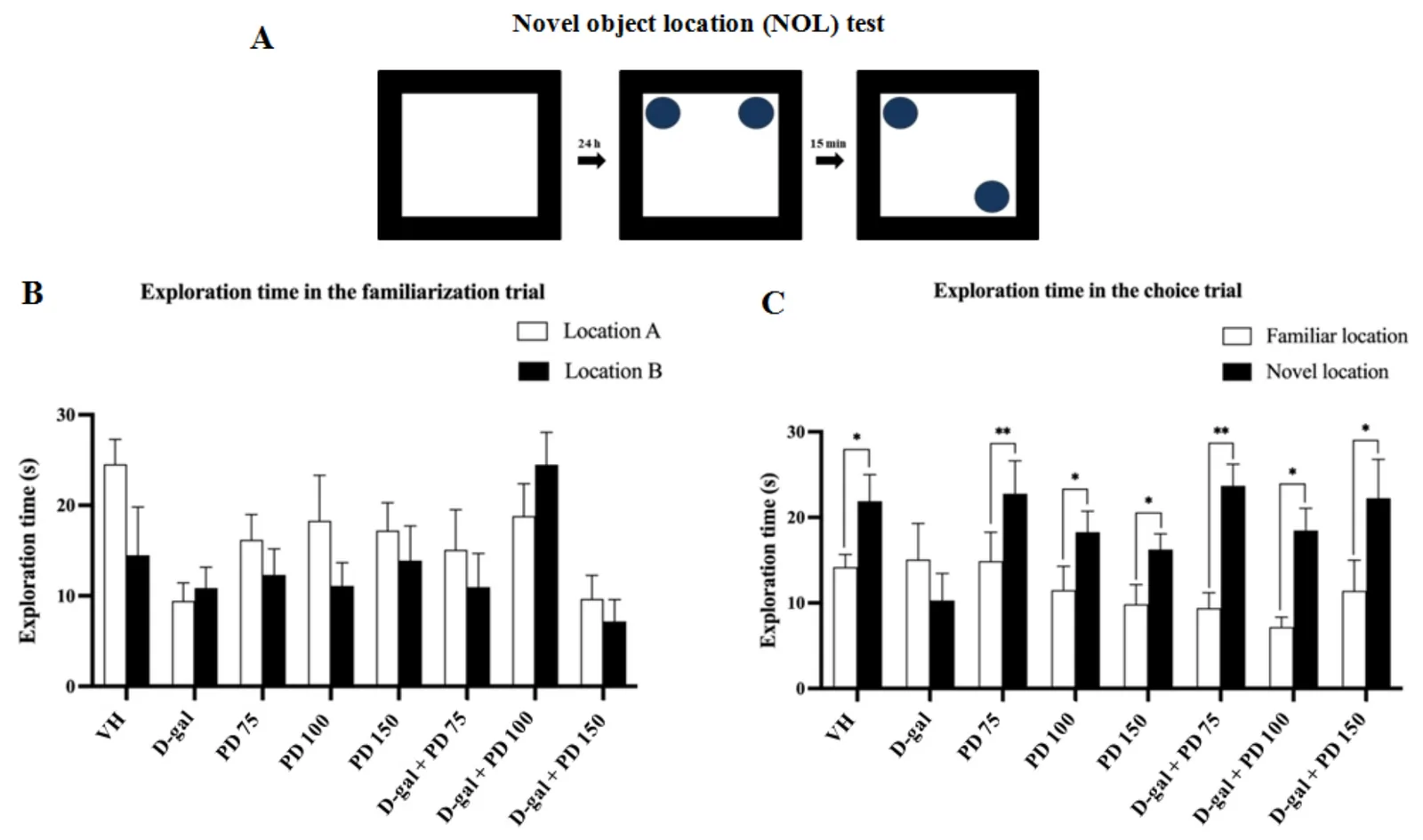
The impact of D-galactose (D-gal) and *Prunus domestica* L. (PD) fruit extract on cognition was examined using the NOL test (**A**). The blue circles represent the objects used in the NOL test. During the familiarization stage (**B**), rats in all groups presented no marked variation during the exploration period for the objects in two locations (*p* > 0.05). During the choice trial (**C**), rats in the vehicle, PD and D-gal + PD groups spent extended time analyzing the novel location when measured against the familiar location (* *p* < 0.05 and ** *p* < 0.01). In contrast, the D-gal group displayed no notably difference in exploratory times between the novel and familiar locations (*p* > 0.05).

**Figure 2:**
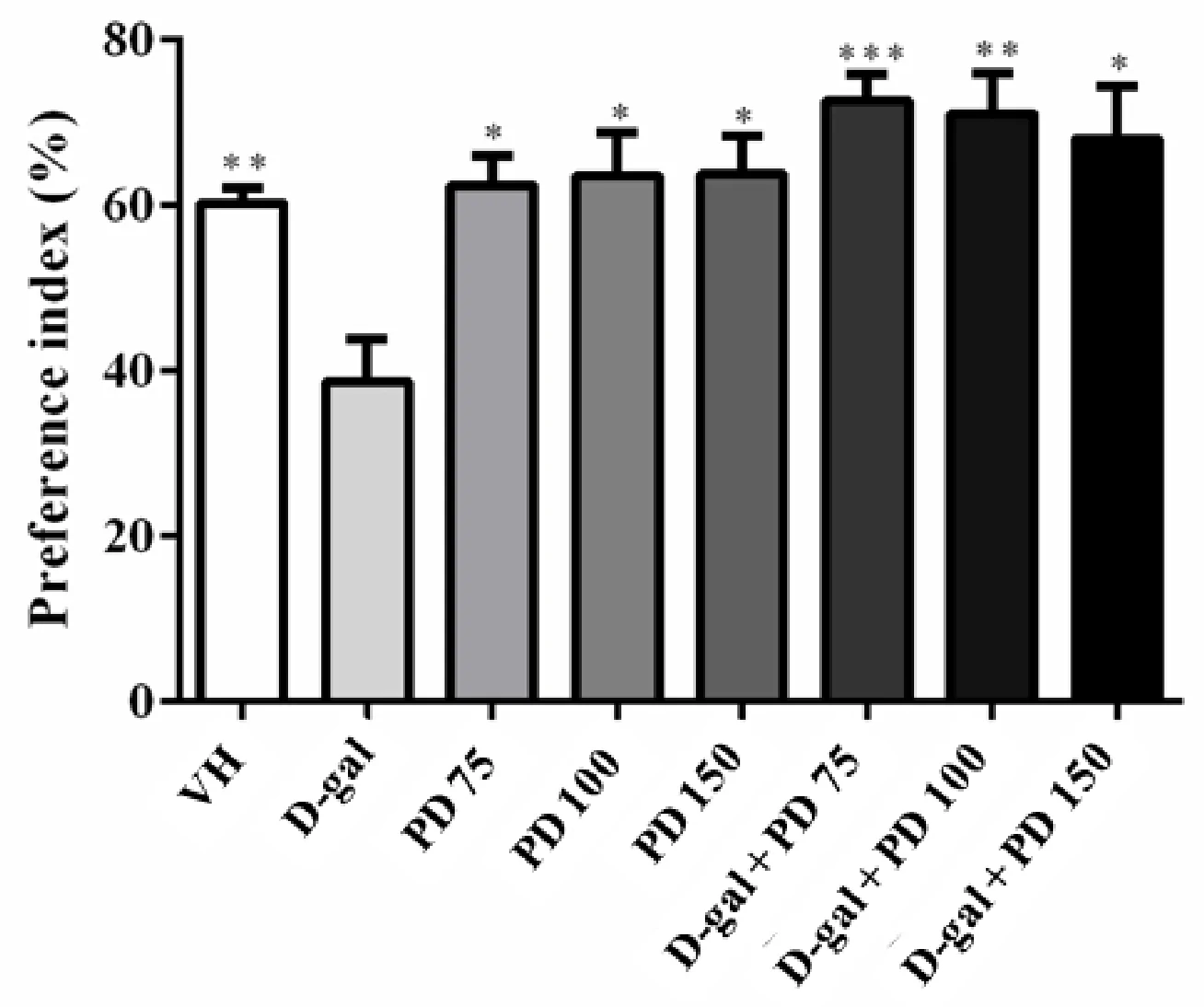
PI during the choice trial of the NOL test. The data on the PIs observed in the vehicle, PD, and D-gal + PD groups were markedly higher than the 50% chance level (* *p* < 0.05, ** *p* < 0.01, and *** *p* < 0.001), while the PI in the D-gal group was below the 50% chance level (*p* > 0.05).

**Figure 3:**
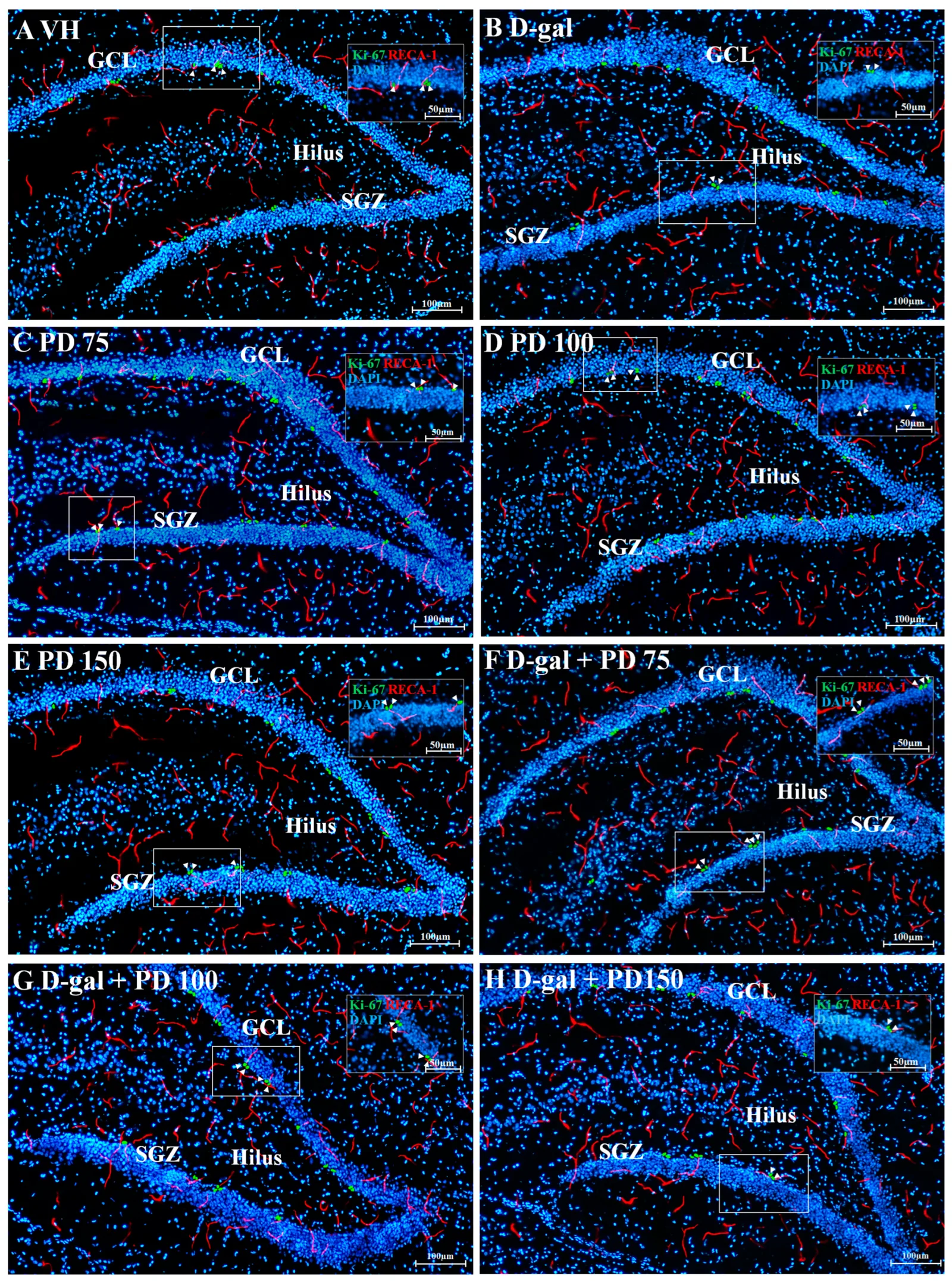
The impact of D-gal and PD fruit extract on Ki-67 (green, indicated by arrowheads) and rat endothelial cell antigen-1 (RECA-1) (red) staining in all groups (**A**–**H**). Abbreviations: SGZ, subgranular zone; GCL, granule cell layer; VH, vehicle; D-gal, D-galactose; PD, *Prunus domestica* L.

**Figure 4:**
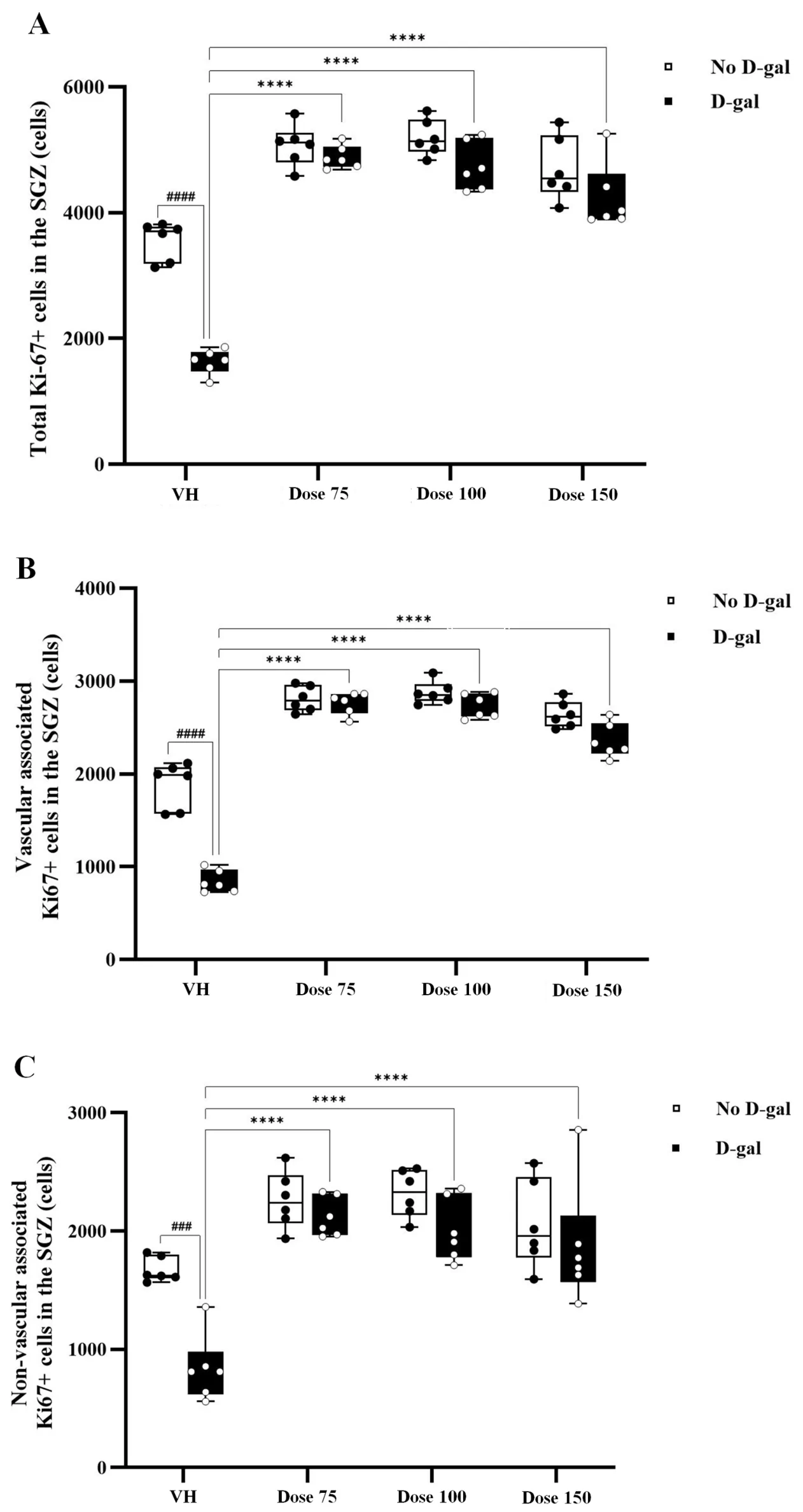
Quantification of Ki-67-stained cells among treatment groups. The total numbers of Ki-67–stained cells (**A**), vascular-associated Ki-67-stained cells (**B**) and non-vascular-associated Ki-67-stained cells (**C**) are shown. The measurements are reported as the mean ± SEM. ^###^ (*p* < 0.001) and ^####^ (*p* < 0.0001) indicate a significant difference compared to the vehicle group, while **** (*p* < 0.0001) indicates a significant difference compared to the D-gal group.

**Figure 5:**
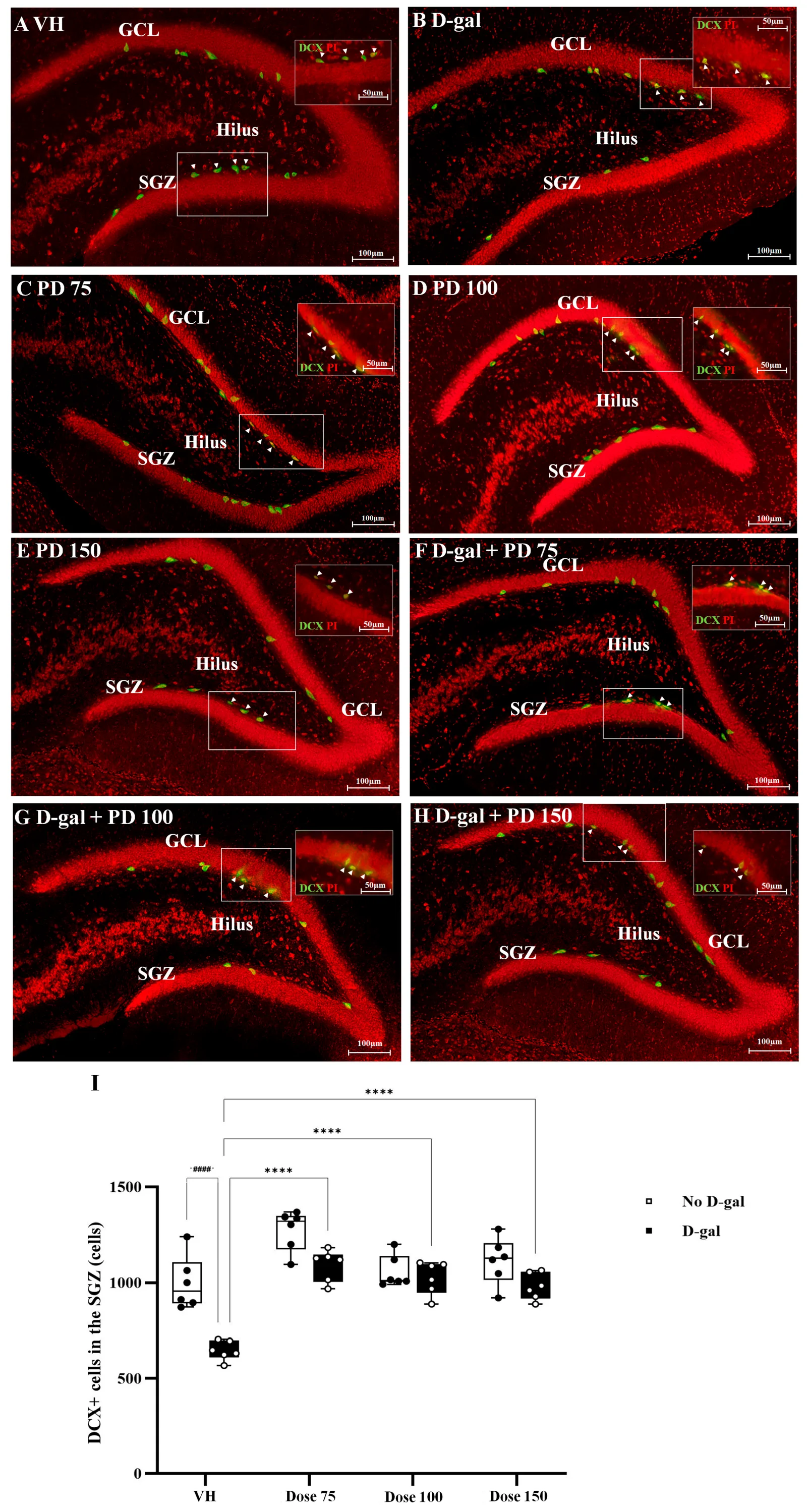
The impact of D-gal and PD fruit extract on DCX-stained cells (green, indicated by arrowheads) in all groups (**A**–**H**). (**I**) illustrates the assessment of DCX determination across all groups. The statistics are demonstrated as the mean ± SEM. ^####^ (*p* < 0.0001) denotes a significant difference compared to the vehicle group, while **** (*p* < 0.0001) denotes a significant difference compared to the D-gal group. Abbreviations: SGZ, subgranular zone; GCL, granule cell layer; VH, vehicle; D, D-galactose; PD, *Prunus domestica* L.

**Figure 6:**
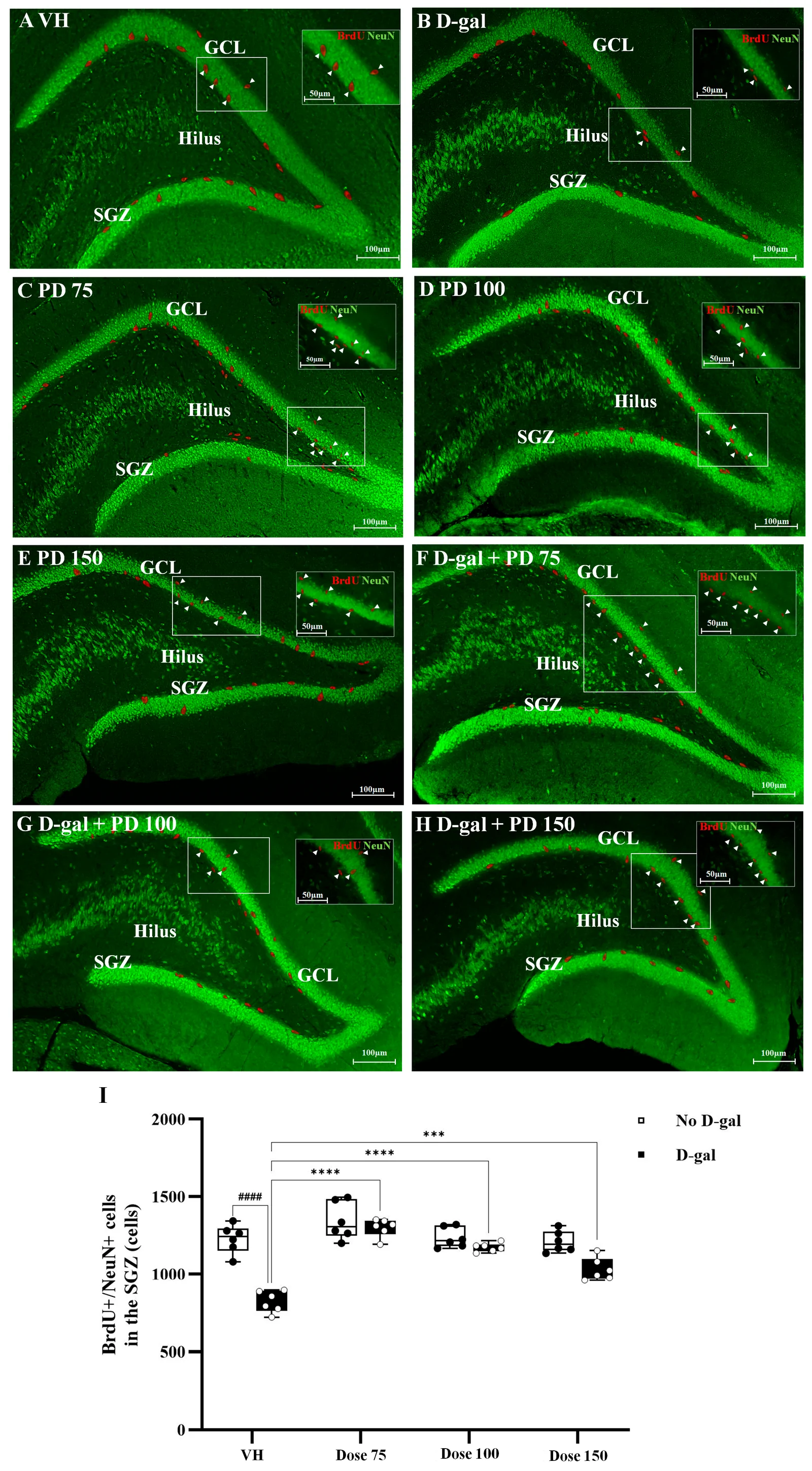
The impact of D-gal and PD fruit extract on BrdU (green, indicated by arrowheads)/NeuN (red)-stained cells in all groups (**A**–**H**). (**I**) illustrates the assessment of BrdU/NeuN determination across all groups. The statistics are demonstrated as the mean ± SEM. ^####^ (*p* < 0.0001) denotes a significant variation compared to the vehicle group, while *** (*p* < 0.001) and **** (*p* < 0.0001) denote a significant variation compared to the D-gal group. Abbreviations: SGZ, subgranular zone; GCL, granule cell layer; VH, vehicle; D, D-galactose; PD, *Prunus domestica* L.

**Table 1:** Distance traveled in the novel object location (NOL) test following treatment.

Groups	Distance Traveled (cm)
VH	5495 ± 1223
D-gal	8435 ± 717.1
PD 75	5351 ± 520.3
PD 100	5584 ± 607.9
PD 150	7985 ± 454.9
D-gal + PD 75	6623 ± 888.8
D-gal + PD 100	6294 ± 825.2
D-gal + PD 150	5792 ± 911.1

Statistics are indicated as the mean ± standard error of the mean (SEM).

**Table 2:** Preference index (PI) of the NOL test following treatment.

Groups	Preference Index (%)
VH	60.02 ± 1.951 **
D-gal	38.59 ± 5.242
PD 75	62.38 ± 3.556 *
PD 100	63.52 ±5.247 *
PD 150	63.78 ±4.611 *
D-gal + PD 75	72.57 ± 3.207 ***
D-gal + PD 100	71.02 ± 4.905 **
D-gal + PD 150	67.99 ± 6.337 *

Statistics are reported as the mean ± SEM. * *p* < 0.05, ** *p* < 0.01, and *** *p* < 0.001 indicate significant differences when measured against a 50% chance.

**Table 3:** Impact of D-gal and PD fruit extract on total, vascular-associated and non-vascular-associated Ki-67-stained cells.

Group	Total Ki-67-Stained Cells	Vascular-Associated Ki-67-Stained Cells	Non-Vascular -Associated Ki-67- Stained Cells
VH	3555 ± 124.23	1883 ± 100.60	1673 ± 42.81
D-gal	1630 ± 80.12 **^####^**	842 ± 48.12 **^####^**	839 ± 114.21 **^###^**
PD 75	5070 ± 134.46	2809 ± 55.75	2261 ± 98.79
PD 100	5195 ± 116.73	2877 ± 48.73	2318 ± 81.89
PD 150	4697 ± 207.03	2640 ± 58.13	2057 ± 151.72
D-gal + PD 75	4883 ± 73.84 ****	2763 ± 47.96 ****	2120 ± 68.53 ****
D-gal + PD 100	4743 ± 157.32 ****	2732 ± 53.33 ****	2012 ± 109.29 ****
D-gal + PD 150	4241 ± 217.75 ****	2358 ± 75.55 ****	1871 ± 208.49 ****

Data are presented as the mean ± SEM (n = 6 per group). *p*-values of (^###^) *p* < 0.001 and (^####^) *p* < 0.0001 indicate a marked variation compared to the vehicle group. A *p*-value of (****) *p* < 0.0001 indicates a marked variation compared to the D-gal group.

**Table 4:** Impact of D-gal and PD fruit extract on developing neuronal cells and neuronal cell survival.

Groups	DCX-Stained Cells	BrdU/NeuN-Stained Cells
VH	997 ± 56.74	1228 ± 37.45
D-gal	645 ± 20.52 **^####^**	821 ± 28.52 **^####^**
PD 75	1275 ± 43.06	1343 ± 49.29
PD 100	1057 ± 34.22	1236 ± 26.51
PD 150	1115 ± 49.99	1209 ± 27.74
D-gal + PD 75	1092 ± 33.48 ****	1300 ± 23.98 ****
D-gal + PD 100	1027 ± 35.28 ****	1173 ± 11.44 ****
D-gal + PD 150	980 ± 28.53 ****	1031 ± 29.82 ***

Data are presented as the mean ± SEM (n = 6 per group). A *p*-value of (^####^) *p* < 0.0001 indicates a marked variation compared to the vehicle group. *p*-values of (***) *p* < 0.001 and (****) *p* < 0.0001 indicate a marked variation compared to the D-gal group. Abbreviations: DCX, doublecortin; BrdU, 5-bromo-2′-deoxyuridine; NeuN, neuronal nuclear protein.

## Data Availability

The data analyzed in this study are available from the corresponding author upon reasonable request.

## Abbreviations

The following abbreviations are used in this manuscript:

AChE	Acetylcholinesterase enzyme
AD	Alzheimer’s disease
BBB	Blood–brain barrier
BDNF	Brain-derived neurotrophic factor
BrdU/NeuN	5-bromo-2′-deoxyuridine/neuronal nuclear protein
CAT	Catalase
CGAs	Chlorogenic acids
COX-2	Cyclooxygenase-2
DAPI	4′,6-diamidino-2-phenylindole
DCX	Doublecortin
DG	Dentate gyrus
D-gal	D-Galactose
GSH-Px	Glutathione peroxidase
H_2_O_2_	Hydrogen peroxide
IL1β	Interleukin 1 beta
Ki-67/RECA-1	Ki-67/rat endothelial cell antigen-1
Malondialdehyde	MDA
MWM	Morris water maze
NO	Nitric oxide
NOL	Novel object location
NPCs	Neural progenitor cells
NSCs	Neural stem cells
pCAMK	Calmodulin-dependent protein kinase
pCREB	Phosphorylated cAMP response element-binding protein
PD	*Prunus domestica* L.
PI	Preference index
SGZ	Subgranular zone
SOD	Superoxide dismutase
TAOC	Total antioxidant capacity
TNFα	Tumor necrosis factor alpha
